# Mesothelin promotes brain metastasis of non-small cell lung cancer by activating MET

**DOI:** 10.1186/s13046-024-03015-w

**Published:** 2024-04-03

**Authors:** Shengkai Xia, Wenzhe Duan, Mingxin Xu, Mengqi Li, Mengyi Tang, Song Wei, Manqing Lin, Encheng Li, Wenwen Liu, Qi Wang

**Affiliations:** 1 Department of Respiratory Medicine, The Second Hospital, Dalian Medical University, Dalian, China; 2 Department of Oncology, Beijing Chest Hospital, Capital Medical University, Beijing, China; 3 Department of Scientific Research Center, The Second Hospital, Dalian Medical University, Dalian, China

**Keywords:** MSLN, NSCLC, Brain metastasis (BM), Blood-brain barrier (BBB), MET

## Abstract

**Background:**

Brain metastasis (BM) is common among cases of advanced non-small cell lung cancer (NSCLC) and is the leading cause of death for these patients. Mesothelin (MSLN), a tumor-associated antigen expressed in many solid tumors, has been reported to be involved in the progression of multiple tumors. However, its potential involvement in BM of NSCLC and the underlying mechanism remain unknown.

**Methods:**

The expression of MSLN was validated in clinical tissue and serum samples using immunohistochemistry and enzyme-linked immunosorbent assay. The ability of NSCLC cells to penetrate the blood-brain barrier (BBB) was examined using an *in vitro* Transwell model and an *ex vivo* multi-organ microfluidic bionic chip. Immunofluorescence staining and western blotting were used to detect the disruption of tight junctions. *In vivo* BBB leakiness assay was performed to assess the barrier integrity. MET expression and activation was detected by western blotting. The therapeutic efficacy of drugs targeting MSLN (anetumab) and MET (crizotinib/capmatinib) on BM was evaluated in animal studies.

**Results:**

MSLN expression was significantly elevated in both serum and tumor tissue samples from NSCLC patients with BM and correlated with a poor clinical prognosis. MSLN significantly enhanced the brain metastatic abilities of NSCLC cells, especially BBB extravasation. Mechanistically, MSLN facilitated the expression and activation of MET through the c-Jun N-terminal kinase (JNK) signaling pathway, which allowed tumor cells to disrupt tight junctions and the integrity of the BBB and thereby penetrate the barrier. Drugs targeting MSLN (anetumab) and MET (crizotinib/capmatinib) effectively blocked the development of BM and prolonged the survival of mice.

**Conclusions:**

Our results demonstrate that MSLN plays a critical role in BM of NSCLC by modulating the JNK/MET signaling network and thus, provides a potential novel therapeutic target for preventing BM in NSCLC patients.

**Supplementary Information:**

The online version contains supplementary material available at 10.1186/s13046-024-03015-w.

## Introduction

Lung cancer is one of the most lethal and aggressive malignancies. Non-small cell lung cancer (NSCLC) accounts for approximately 85% of the total incidence of lung cancer and adenocarcinoma is the most common pathological type of NSCLC [1]. Approximately 50% of NSCLC patients eventually develop brain metastasis (BM), and the median survival of lung cancer patients with brain metastasis is only 4-6 months [2, 3]. The identification of oncogenic driver gene alterations has refined the staging of NSCLC, and targeted drugs against these oncogenes have dramatically improved the treatment outcomes. *MET* is one of the most common oncogenes in NSCLC. It encodes MET, also known as hepatocyte growth factor receptor (HGFR), which is characterized as a high affinity transmembrane receptor tyrosine kinase (RTK). Its activation in a phosphorylated state (p-MET) has been shown to play a critical role in NSCLC tumor progression and invasion [4]. Inhibition of p-MET significantly reduces the cellular activity and aggressiveness of NSCLC cells, thereby significantly reducing the incidence of BM [5]. Capmatinib, a selective MET inhibitor which was approved by the Food and Drug Administration (FDA) in 2020, has shown activity in malignancies with MET activation including the advanced NSCLC [6, 7]. It is worth noting that crizotinib, which was approved by the FDA in 2016, although primarily indicated for the anaplastic lymphoma kinase (ALK)-rearranged lung cancer in clinical application [8], also has an inhibitory effect on MET. However, their specific therapeutic effects on NSCLC BM need to be further clarified.

The blood-brain barrier (BBB), which is composed of a network of closely opposed endothelial cells in the cerebral capillaries and characterized by the presence of continuous tight junctions (TJs), serves as a barrier between the peripheral circulatory system and neural tissue [9]. The “opening” of TJs by tumor cells in order to penetrate the BBB is the decisive rate-limiting step in the development of BM. However, the underlying mechanisms remain largely unknown.

Mesothelin (MSLN) is a tumor differentiation antigen that has recently been found to be overexpressed in many types of solid tumors, including lung cancer [10–12]. Abnormal MSLN expression promotes tumor development by inducing tumor cell proliferation, anti-apoptosis and metastasis [13–15]. Targeted therapies against MSLN have showed antitumor activaties in MSLN-positive tumors [16, 17]. In lung cancer, MSLN promotes epithelial-mesenchymal transition (EMT) and stemness of tumor cells, tumorwhich may facilitate the occurrence of BM [18]. Recently, MSLN-specific cellular immune responses were identified in the blood of patients with BM and regarded as a predictor for survival, which indicates the involvement of MSLN in BM [19].

In the present study, we determined the high expression of MSLN and its promotive role in BM of NSCLC, and revealed the underlying mechanism that MSLN promotes tumor cell extravasation across the BBB by facilitating the expression and activation of MET through the c-Jun N-terminal kinase (JNK) signalling pathway. Our results also showed that targeting MSLN with anetumab or MET with crizotinib or capmatinib effectively prevents the development of BM *in vivo*.

## Methods

### Cell culture and drugs

Human bronchial epithelial cells (16HBE cells), human lung fibroblasts (HFL1 cells), human monocyte cells (THP-1 cells), normal bronchial epithelial cells (BEAS-2B cells) and lung cancer cell lines (H1299, H2030, PC9, H1975, H460, and H226 cells) were purchased from the Chinese Academy of Medical Sciences (Beijing, China). The brain metastatic lung cancer cell line H2030-BrM and PC9-BrM was generated by injecting H2030 cells and PC9 cells into the left ventricle of immunodeficient mice and isolating the metastatic cells from harvested areas of brain metastases. Human lung microvascular endothelial cells (hPMECs), human brain microvascular endothelial cells (hBMVECs) and human astrocytes (HA-1800) were purchased from Sciencell (Sciencell, USA) and cultured in the appropriate medium recommended by the manufacturer. The above cell types were cultured at 37°C in humidified air with 5% CO_2_. The different cell types were authenticated by short tandem repeat profiling and tested for mycoplasma contamination.

JNK-IN-8 (HY-13319), Crizotinib (HY-50878), Anetumab (HY-P99352) and Capmatinib (HY-13404) were purchased from MedChemexpress (USA).

### SDS-PAGE and western blot analysis

Cells were lysed in a mixture of radioimmunoprecipitation assay (RIPA) protein lysis buffer (Thermo Scientific) containing the protease inhibitor phenylmethylsulfonyl fluoride (PMSF, Beyotime) and a phosphatase inhibitor cocktail (Sigma-Aldrich) at 4°C for 45 min. The BCA Protein Quantification Kit (Thermo Fisher Scientific) was used to measure protein concentrations. Proteins (30 μg/lane) were separated on 10% sodium dodecyl sulfate (SDS)-polyacrylamide gel electrophoresis (PAGE) gels and transferred to nitrocellulose membranes (Millipore, USA). Each membrane was blocked with protein-free rapid blocking buffer (EpiZyme, Shanghai) for 15 min and then incubated overnight at 4°C with the following antibodies: anti-E-cadherin (1:500, 13-1700, Invitrogen), anti-N-cadherin (1:200, sc-393933, Santa Cruz Biotechnology), anti-Slug (1:500, ab27568, Abcam), anti-matrix metalloproteinase 7 (MMP7, 1:1000, ab205525, Abcam), anti-MSLN (1:3000, ab133489, Abcam), anti-glyceraldehyde phosphate dehydrogenase (GAPDH, 1:5000, 10494-1-AP, Proteintech), anti-cleaved poly(ADP-ribose) polymerase (PARP, 1:1000, #5625, Cell Signaling Technology), anti-cleaved caspase-3 (1:1000, #9661, Cell Signaling Technology), anti-vascular endothelial (VE)-cadherin (1:1000, ab205336, Abcam), anti-junctional adhesion molecule (JAM)-A (1:1000, ab269948, Abcam), anti-claudin 5 (1:1000, ab131259, Abcam), anti-phosphorylated (p)-MET (1:5000, ab68141, Abcam), anti-MET (ab51067, 1:5000, Abcam), anti-p-JNK (1:1000, #4668, Cell Signaling Technology), and anti-t-JNK (1:1000, #9252, Cell Signaling Technology). The next day, the membranes were washed with Tris-buffered saline with Tween 20 (TBST) for 8 min at room temperature twice and then incubated with secondary antibodies (1:5000, SA00001-2, Proteintech; 1:5000, SA00001-1, Proteintech) for 1 h at room temperature. The membranes were then washed three times with TBST for 8 min each, followed by scanning and visualization of the immunoreactivity by enhanced chemiluminescence (ECL, Advansta). Protein expression in three independent experiments was quantified using ImageJ software (National Institutes of Health, USA).

### Clinical samples

Tissue samples from 70 NSCLC patients and serum samples from 154 participants (untreated patients with NSCLC or primary brain tumor and healthy volunteers) were collected from the Second Affiliated Hospital of Dalian Medical University, Dalian, China. The diagnosis of NSCLC and primary brain tumor was confirmed by pathology (surgical resection and/or biopsy). All patients with advanced NSCLC and primary brain tumors completed baseline brain MRI examinations at the time of initial diagnosis and before receiving anti-tumor therapy. Written informed consent was obtained from all participants. This study was approved by the Ethics Review Committee of the Second Hospital of Dalian Medical University (2020-020). In addition, the information of 478 lung cancer patients used by the GEPIA database (http://gepia.cancer-pku.cn/) was obtained from the TCGA database (Table S1) [20].

### Enzyme-linked immunosorbent assay (ELISA)

Target protein concentrations were measured using a Human MMP7 ELISA Kit (Elabscience) and a Human MSLN ELISA Kit (Omnimabs). We followed the manufacturer’s instructions and measured the optical density (OD) of the solution in each well at 450 nm. Finally, the protein concentration in each sample was obtained by comparison with the standard curve.

### Immunohistochemistry (IHC) staining and scoring

Surgical specimens were embedded in paraffin and sectioned for IHC analysis. The tissue sections were dewaxed, hydrated, and rinsed in running water for 10 min. The sections were then soaked in boiling sodium citrate antigen repair solution for 20 min before addition of endogenous peroxidase blocker followed by dropwise addition of normal goat serum working solution for blocking. Solutions of anti-MSLN antibody (1:100, Proteintech, 66404-1-Ig) and anti-MET antibody (1:150, Proteintech, 25869-1-AP) were added for incubation overnight at 4°C. The following day, biotin-labeled secondary antibody was added dropwise followed by dropwise addition of horse radish peroxidase(HRP)-labeled streptavidin working solution. The tissue sections were then stained with diaminobenzidine(DAB), rinsed with tap water, stained with hematoxylin for 20s, and rinsed again before being dehydrated, cleared, and sealed. IHC images were quantitatively assessed and automatically scored using the IHC Profiler open source plugin[21].

### Establishment of stable overexpression and knockdown cell lines

PC9 and PC9-BrM cells were transfected with viral vectors for MSLN overexpression and knockdown, respectively, according to the manual for lentivirus use (Shanghai Genechem Co., Ltd.), and the overexpressed or knockdown cells were screened with puromycin (1 µg/ml) for 1 week. The target sequences of MSLN shRNA1 and shRNA2 were 5'- GGAUGAGCUCUACCCACAATT-3' and 5'-CUUGCUUUCCAGAACAUGATT-3', respectively. PC9-BrM cells were transfected with MET-specific small interfering RNAs (siRNAs) (siRNA-1: 5'- GCCUGAAUGAUGAUGACAUUCUTT-3' and siRNA-2:5'- GCUGGUGGCACUUUACUUATT -3') using Lipofectamine 2000 or control siRNA (GenePharma) for 48 h. MSLN knockdown in PC9-BrM cells (PC9-BrM-SH2) was achieved by transfection of the cells with MET plasmid or negative control plasmid using Lipofectamine 2000 for 48 h, after which the cells were screened with G418 for 1 week. The efficiency of overexpression or knockdown was assessed by western blotting.

### Wound healing assays

Cells were inoculated in 6-well plates, and once they reached confluency, wounds were created using a sterile 100-μl pipette tip. The cells were washed in suspension with phosphate-buffered saline (PBS) and imaged. After 24 h of incubation in serum-free medium, the healing process of cells migrating to cover the wound area was observed microscopically and imaged. Cellular wound healing rates were analyzed using ImageJ software.

### Transwell migration and invasion assays

Cell suspensions with a cell density of 2×10^6^ cells/ml in 200 µl of serum-free medium were added to Transwell chambers. For invasion experiments, the Transwell membrane was wrapped in advance with a matrix gel (BD, USA). The lower chamber was then spiked with medium containing 20% fetal bovine serum and incubated for 24 h. The cells were then fixed in 4% paraformaldehyde for 20 min and stained with crystal violet for 20 min. The stromal gel and cells were removed from the Transwell chamber layer with a cotton swab and photographed under a microscope to observe and count the cells. The data were analyzed using ImageJ software.

### Trans-endothelial assays

hBMVECs (1×10^5^) and HA1800 cells (1×10^5^) were inoculated in the upper and bottom wells of a Transwell chamber and cultured until complete monolayers had formed. Brain metastatic cells (4×10^5^) in medium containing 1% serum were inoculated in the top inserts, and 500 µl of medium with 20% serum was added to the bottom chamber. After 24 h, the green fluorescent protein (GFP)-labeled BM cells that had invaded via the membrane were photographed on a fluorescent microscope for counting.

### Trans-BBB assays on a microfluidic chip

A bionic multi-organ microfluidic chip that allows real-time visual monitoring of the entire BM process was fabricated as previously described [22]. Briefly, tumor cells were edited to stably express GFP, while hBMVECs were labeled red with the Cell Tracker^TM^ CM-Dil dye (Invitrogen, USA) according to the manufacturer’s instructions. After the biomimetic “lung” organ and “brain” organ were constructed, tumor cells were introduced to the upstream “lung” organ to allow the occurrence of BM. The trans-BBB events were then observed using on inverted fluorescent microscope. The observation starting time was designated as the time when the first cell reached the downstream vascular channel along with the fluid, and the images were captured after 36 h.

### Generation of conditioned medium

Tumor cells were incubated in serum-free medium on 6-well plates for 24 h. After 24 h, tumor cell supernatant samples were centrifuged and filtered to remove cellular debris. The collected conditioned medium was then stored at -80℃ until further use.

### Immunofluorescence (IF) staining

For immunofluorescent staining, hBMVECs were washed three times with PBS, fixed in 4% paraformaldehyde, and permeabilized in 0.1% Triton X-100 solution (Sigma, USA) for 10 min. For blocking, 3% Bovine serum albumin (BSA) solution was added for 30 min, and then cells were incubated with primary antibody (anti-JMA-A, Abcam; anti-VE-cadherin and anti-claudin 5, Invitrogen; 1:50 dilution) overnight at 4°C. After three washes with PBS, the cells were incubated in solution of fluorescein isothiocyanate (FITC)-labeled secondary antibody (1:100 dilution; Proteintech, USA) at room temperature. Cell nuclei were stained with 1 µg/ml 4',6-diamidino-2-phenylindole (DAPI) (1:1000 dilution; Sigma, USA) for 10 min at room temperature. Images were obtained using a confocal microscope (Leica TCS SP5II, Germany).

### Quantitative reverse transcription-polymerase chain reaction (qRT-PCR)

Total RNA was extracted from different groups of cells and reverse transcribed to cDNA using the One-Step gDNA Removal and cDNA Synthesis SuperMix (AT311, Transgen Biotech, Beijing, China) according to the manufacturer’s protocol. The relative levels of MSLN mRNA transcripts, MET mRNA transcripts, and GAPDH transcripts were quantified by qRT-PCR using Top Green qPCR SuperMix (AQ131, Transgen Biotech) and the following specific primers. The primer sequences were: h-MSLN-F 5'-CTGGAAGCCTGCGTGGAT-3′ and h-MSLN-R 5'-CCAGGTGCTGGATCACAGACT-3′; h-MET-F 5'- TCCAGGCAGTGCAGCATGTA-3′ and h-MET-R 5'-TCAAGGATTTCACAGCACAGTGA-3′; h-MMP7-F 5'- AGAGATCCCCCTGCATTTCA-3′ and h-MMP7-R 5'- GCCCATCAAATGGGTAGGAGT-3′;

h-GAPDH-F 5'-CATGAGAAGTATGACAACAGCCT-3′ and h GAPDH-R 5'-AGTCCTTCCACGATACCAAAGT-3′. All data were analyzed by the 2-ΔΔCt method.

### Animal study

Four-week-old female BALB-c-nu mice were purchased from Beijing Vital River Laboratory Animal Technology Co., Ltd. (China). The PC9 cell line stably expressing GFP-luciferase fusion protein was constructed [22], cultured and collected in a cell suspension of 1×10^7^ cells/ml. After the mice were anesthetized, 100 µl of cell suspension was injected into the left ventricle of each mouse. After retro-orbital injection of D-Luciferin (150 mg/kg body weight; Promega, USA) at the indicated time points, images of mouse tumor metastases were acquired using the IVIS Spectrum Xenogen instrument (PerkinElmer, USA). *In vivo* imaging software (version 2.50) was used to analyze the bioluminescence images. All animal experiments were performed in accordance with a protocol approved by the Animal Protection and Use Committee of Dalian Medical University.

### BBB leakiness assay

Mice were injected in the tail vein with 100mg/kg Texas Red dextran (70,000 MW, Thermo Fisher Scientific, D1864). After 3 hours, mice were injected in the tail vein with 10 mg/kg DyLight 488-Lycopersicon Esculentum Lectin (LEL) (Thermo Fisher Scientific, L32470). After 10 minetes, each mouse was anaesthetised and perfused with ice PBS until there was no blood, followed by 4% paraformaldehyde for 3-5 min. Brain tissue was extracted and immersed in 30% sucrose overnight. Tissue cryosections with 6 µm thick were stained with DAPI (Solarbio) and images were obtained with an Leica TCS SP5II confocal microscopy (Leica). Three random areas of each section were collected and three sections of each brain were examined.

### Statistical analysis

The data are expressed as the mean ± standard deviation (SD). The data were plotted using GraphPad Prism 8.0 software and then statistically analyzed using Statistical Package for the Social Sciences (SPSS) 19.0 software. To identify statistical differences between groups, the data were compared among experimental groups using analysis of variance (ANOVA), t-test or chi-square test. Statistical significance was defined by *P*<0.05.

## Results

### Increased MSLN expression correlates with BM of NSCLC

**Fig. 1:**
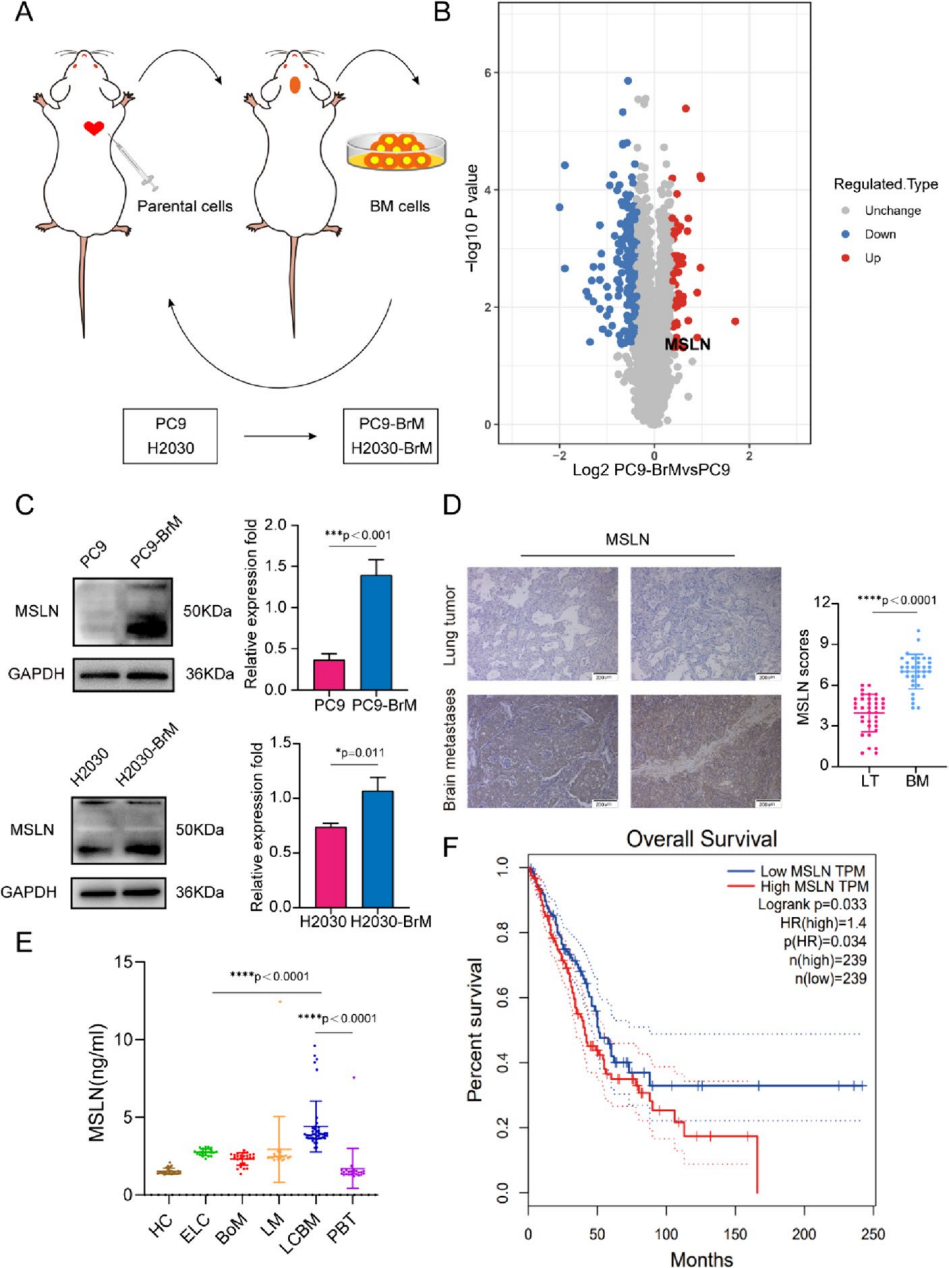
MSLN expression is increased in NSCLC patients with BM.** A** Schematic illustration of the selection process of brain metastasis(BM) derivatives in mice. Parent cells PC9 and H2030 were inoculated into the left ventricle of nude mice to isolate and collect tumor cells with BM. The selection process was carried out twice, and the high-brain metastatic subpopulation (PC9-BrM and H2030-BrM cell lines) were collected. **B** Differential protein volcano map between PC9-BrM cells and PC9 cells. **C** Western blot analysis showed that PC9-BrM and H2030-BrM cells with high metastatic activity had higher MSLN protein levels. **D** Representative images and quantification analysis of MSLN staining in primary lung tumor (LT, *n*=36) and NSCLC-derived brain metastases (BM, *n*=34) surgical specimens. (scale bar, 200 μm) . **E** ELISA detection of MSLN expression in serum of all patients and control groups. HC, healthy controls (*n*=24). ELC, early-stage NSCLC (*n*=22). BoM, lung cancer with bone metastasis (*n*=23). LM, lung cancer with live metastasis (*n*=20). LCBM, lung cancer with brain metastasis (*n*=42). PBT, primary brain tumor (*n*=23). **F** Kaplan-Meier analysis of the overall survival of 478 lung cancer patients in the GEPIA database. (Data are presented as mean ± SD)

To identify proteins potentially involved in the BM of NSCLC, we employed two NSCLC cell lines, PC9 (EGFR^Dexon19^ mutation) and H2030 (K-ras^G12C^ mutation) cells, to develop a high-brain metastatic subpopulation (PC9-BrM and H2030-BrM, Fig. 1A, Fig. S1A) and performed further proteomics analysis in PC9 and PC9-BrM cells to characterize the protein expression profile found in BM in our previous work[22, 23]. Our results showed that the expression of MSLN was significantly up-regulated in the protein profiling (Fig. 1B), and the increased MSLN expression was verified by western blotting in both PC9-BrM and H2030-BrM cells compared to the respective parental cells (Fig. 1C). We also measured MSLN expression in a normal human bronchial epithelial cell line (BEAS2B) and in five NSCLC cell lines and found that MSLN was not detected in non-cancerous BEAS2B cells but was clearly expressed in the NSCLC cell lines characterized by preferential BM capacity, such as in PC9, H460 and H226 cells (Fig. S1B) [24, 25].

We next examined the expression of MSLN in clinical samples. 34 brain metastatic tumors were obtained from NSCLC BM patients while 36 primary lung tumors were obtained from patients with early stage NSCLC. We performed IHC for MSLN and found that MSLN expression was higher in the BM tissues than in the neoplastic tissues in situ (Fig. 1D, Table S2). Recent studies have indicated that the presence of soluble MSLN in serum samples may also be a potential serum biomarker for malignancies [26]. Hence, we also detected the levels of MSLN in serum samples. Serum samples were collected from an untreated patients cohort (*n*=154) including 107 patients with NSCLC, 23 with primary brain tumors (PBT) and 24 healthy controls (HC). The 107 NSCLC patients included 22 cases of early lung cancer (ELC), 23 cases with bone metastasis (BoM), 20 cases with liver metastasis (LM) and 42 cases with lung cancer brain metastasis (LCBM). The clinicopathological imaging characteristics of the cohort are shown in Table S3. We found that the average MSLN level of the ELC group was higher than that of the HC group. Further comparison between the LCBM group and the ELC group showed that the MSLN level in the LCBM group was higher than that in the ELC group (Fig. 1E). In the analysis of the correlation between serum MSLN expression and clinicopathological imaging characteristics of lung cancer patients with BM, it was found that the serum level of MSLN was significantly correlated with smoking history, BM maximum diameter, meningeal metastasis, number of primary lung lesions, pleural effusion, and epidermal growth factor receptor (EGFR) mutation status (Table S4). Moreover, analysis of the GEPIA public database showed that high MSLN expression in NSCLC patients was significantly associated with low survival (*n*=478, Fig. 1F). Together these findings indicate that MSLN is involved in malignant progression of lung cancer and may play an important role in promoting BM in NSCLC.

### MSLN promotes the migration and invasion of NSCLC brain metastatic cells in vitro

**Fig. 2:**
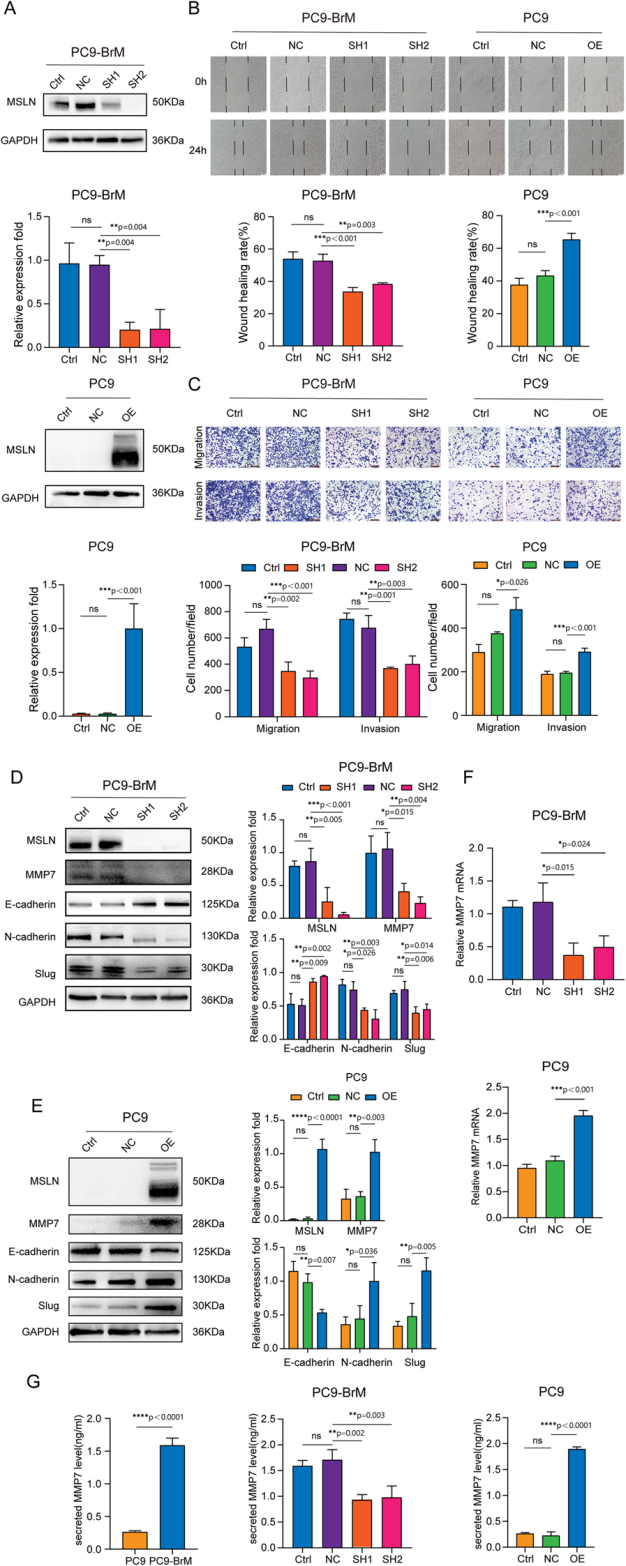
MSLN promotes the migration and invasion of NSCLC cells *in vitro*. A Representative images and quantitative results of western blotting showing MSLN expression after transfection of PC9-BrM and PC9 cells with lentivirus. **B** The effect of MSLN expression on the migration capacity in PC9-BrM and PC9 cells assessed in a wound-healing assay (scale bar, 100 µm).** C** Transwell migration and invasion assays to determine the effect of altered MSLN expression on the migration and invasion of NSCLC cells (scale bar, 200 µm). **D, E** Western blot analysis of E-cadherin, N-cadherin, Slug and MMP7 expression in PC9-BrM cells and PC9 cells after alteration of MSLN expression. **F-G** The expression of MMP7 by PC9 cells and PC9-BrM cells was detected by qRT-PCR (F) and ELISA (G). (PC9-NC, PC9 cells transfected with negative control plasmid. PC9-OE, PC9 cells transfected with MSLN plasmid. PC9-BrM-NC, PC9-BrM cells transfected with negative control shRNA. PC9-BrM-SH1, PC9-BrM cells transfected with MSLN-targeted shRNA1. PC9-BrM-SH2, PC9-BrM cells transfected with MSLN-targeted shRNA2. Data are presented as mean ± SD, ns, no significance)

It is well known that tumor cells undergo EMT to become aggressive and migratory, and this is an important event in tumor metastasis and tumor progression [27]. Our previous study found that the brain metastatic cell line PC9-BrM exhibits a mesenchymal-like phenotype and strong ability to migrate and invade [22], as do H2030-BrM cells as demonstrated in this study (Fig. S2). Some studies have concluded that MSLN plays an important role in tumor migration and invasion [28, 29]. Hence, we hypothesized that MSLN plays a role in the process of BM of NSCLC. In our experiments, knockdown of MSLN significantly reduced the migratory and invasive capacity of brain metastatic NSCLC cells (PC9-BrM and H2030-BrM), whereas overexpression of MSLN increased the migratory and invasive capacity of the parental cells (PC9 and H2030, respectively), as evidenced by the wound healing and Transwell assays (Fig. 2A-C, Fig. S3). These results suggest that MSLN significantly promotes the migration and invasion of NSCLC brain metastatic cells. In addition, we examined the expression of EMT markers by western blotting and found that knockdown of MSLN reversed the mesenchymal phenotype of PC9-BrM cells based on increased E-cadherin expression and decreased N-cadherin and Slug expression (Fig. 2D), while overexpression of MSLN facilitated EMT of PC9 cells (Fig. 2E).

Because the expression of matrix metalloproteinase family members (MMPs) is essential for tumor progression and metastasis [28–30], we further analyzed whether MMPs are involved in the MSLN-induced enhanced aggression of brain metastatic cells. We found that MMP7, rather than the more widely studied MMP2/9, was regulated by MSLN. The level of cellular and secretory MMP7, as determined by Western blotting, qRT-PCR and ELISA, was significantly decreased by MSLN deletion in PC9-BrM cells and was increased by MSLN overexpression in PC9 cells (Fig. 2D-G). In summary, our data indicate that MSLN plays a promotive role in the enhanced migration and invasion of NSCLC brain metastatic cells.

### MSLN helps brain metastatic cells penetrate the BBB by degrading inter-endothelial TJs

**Fig. 3:**
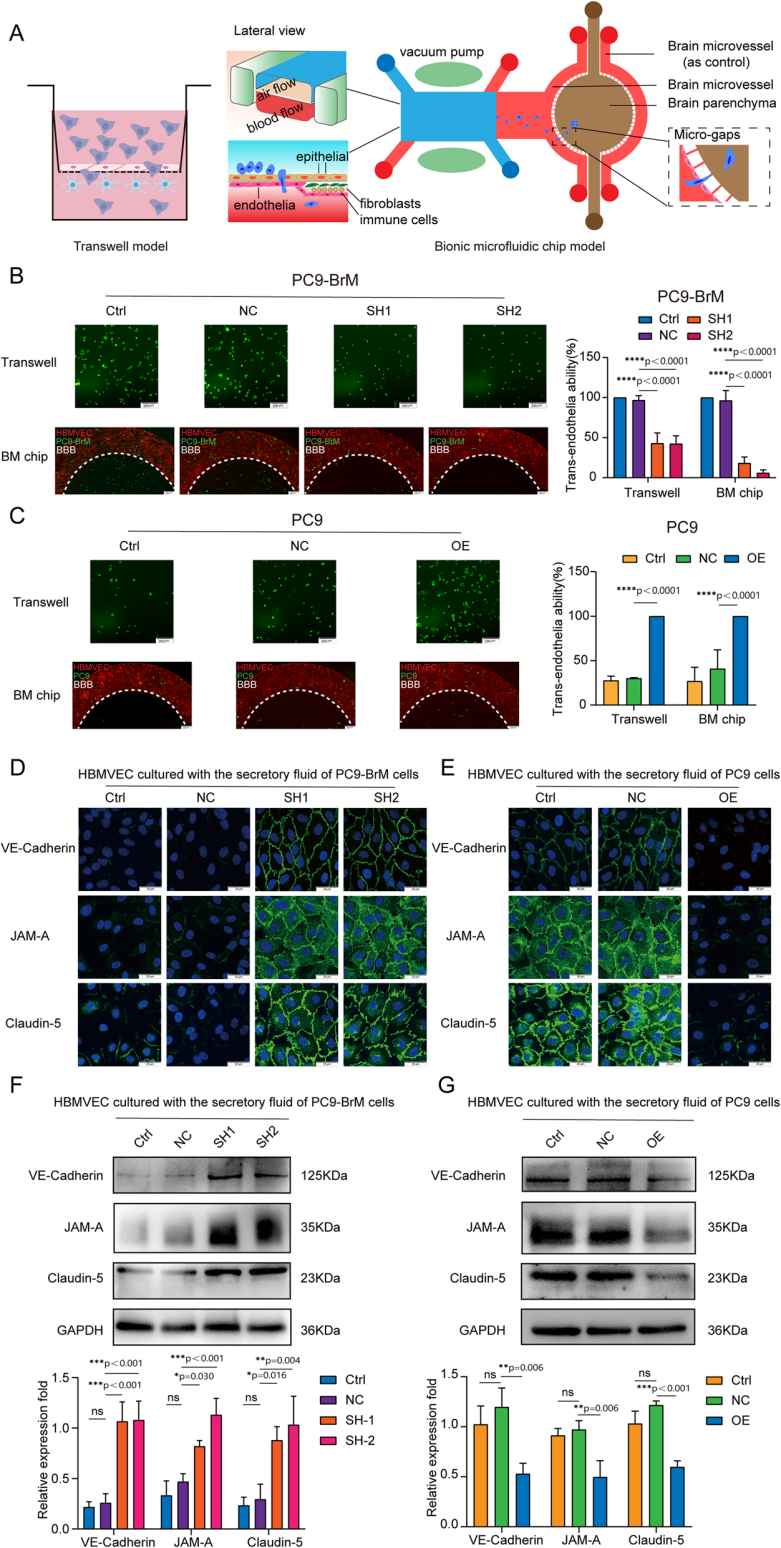
MSLN promotes NSCLC cells penetration of endothelium by promoting cleavage of endothelial TJ proteins. **A** Schematic diagrams of a classic *in vitro* blood-brain barrier (BBB) model and the multi-organ microfluidic chip. **B**, **C** Representative images of the ability of tumor cells to penetrate the BBB in the Transwell assay and the chip (scale bar, 200 µm). **D**, **E** Representative confocal microscopy images showing the distribution of VE-cadherin, JAM-A and claudin-5 in a hBMVEC monolayer (scale bar, 20 µm). **F, G** Western blot analysis of VE-cadherin, JAM-A and claudin-5 expression in hBMVECs after treatment with conditioned medium from the indicated tumor cells. (TJ, tight junction. PC9-NC, PC9 cells transfected with negative control plasmid. PC9-OE, PC9 cells transfected with MSLN plasmid. PC9-BrM-NC, PC9-BrM cells transfected with negative control shRNA. PC9-BrM-SH1, PC9-BrM cells transfected with MSLN-targeted shRNA1. PC9-BrM-SH2, PC9-BrM cells transfected with MSLN-targeted shRNA2. Data are presented as mean ± SD, ns, no significance)

The BBB can restrict the invasion of many pathogens, and therefore, the crossing of the BBB by tumor cells is a critical step in BM [31]. We found that the highly brain metastatic cells were more capable of penetrating an endothelial cell layer than were the parental cells (Fig. S4A) [22]. To further investigate the role of MSLN in BM, we examined the trans-endothelial cell migration ability of brain metastatic cells with or without MSLN interference, using an *in vitro* BBB model and an *ex vivo* bionic BBB microfluidic chip model established previously [22]. The *in vitro* BBB model was established using Transwell chambers coated with human brain microvascular endothelial cells (hBMVECs) and primary human astrocytes (HA-1800), while the *ex vivo* bionic BBB model was established on a well-designed microfluidic chip where HA-1800 cells were introduced into the brain parenchyma chamber and hBMVECs were introduced into the vascular channels for co-culture with HA-1800 cells under the dynamic flow shear force (Fig. 3A). The results showed that silencing MSLN resulted in a significant decrease in the ability of brain metastatic NSCLC cells to penetrate the endothelium (Fig. 3B, Fig. S4B). Consistently, trans-endothelial migration was significantly increased among parental cells overexpressing MSLN (Fig. 3C, Fig. S4C). These results suggest that MSLN helps metastatic NSCLC cells to cross the BBB. TJs, the key structures that maintain the barrier function of the BBB, mainly consist of ocludin, junctional adhesion molecules(JAMs), claudins, zonula occludens (ZO), and calmodulin (VE-cadherin), which form a junctional complex to maintain the stability of the barrier. Disruption of the junctional complex leads to the loss of cell-cell contacts and the formation of cellular gaps, which creates the opportunity for tumor cells to cross the BBB [31].

We next investigated whether MSLN in NSCLC cells affected the expression of these junctional complex proteins by hBMVECs. We treated hBMVEC monolayers for 24 h with conditioned medium obtained over a 24 h period from the indicated tumor cells and then observed the distribution of VE-cadherin, JAM-A and claudin-5 expression by immunofluorescence imaging. The image results showed that the distributions of VE-cadherin, JAM-A and claudin-5 became discontinuous and vastly diminished in hBMVEC monolayers treated with conditioned medium derived from tumor cells with relatively high expression of MSLN (Fig. 3D, E). We further quantified the expression of TJ complex proteins by western blotting. Consistent with the immunofluorescence observations, we found that conditioned medium from tumor cells with MSLN knockdown did not induce as much degradation of the cadherin, JAM-A and claudin-5 expression patterns among hBMVEC monolayers (Fig. 3F). Conversely, the degradation of these endothelial junction proteins was significantly increased after treatment of hBMVECs with conditioned medium from MSLN-overexpressing parental cells (Fig. 3G). These results indicate that MSLN promotes the ability of brain metastatic cells to penetrate the BBB by destroying TJ complexes.

### Disruptive effect of MSLN on TJs is dependent on MET expression

**Fig. 4:**
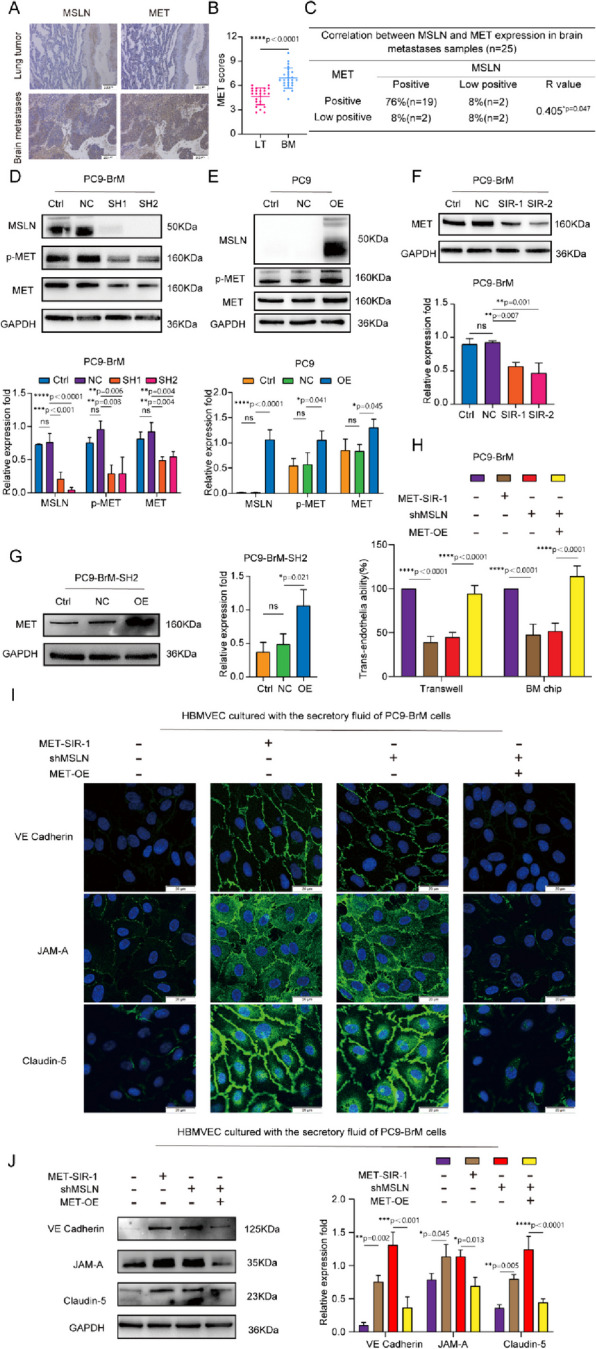
The effect of MSLN on NSCLC penetration of the BBB is dependent on MET. **A,B** Representative images and quantification analysis of MSLN and MET staining in primary lung tumor (LT, *n*=25) and lung cancer-derived brain metastases (BM, *n*=25) surgical specimens (scale bar, 200 μm). **C** Correlation of MSLN and MET protein expression in BM surgical specimens. **D, E** Representative western blot images showing the expression of MSLN, p-MET and MET in the indicated cells. **F, G** Representative images and quantitative results of western blotting for MET expression in tumor cells after transfection. **H** Effect of MET on the ability of NSCLC cells to penetrate the endothelium. **I, J** After treatment of hBMVECs with conditioned medium from the designated tumor cells for 24 h, western blot analysis and immunofluorescence detection showed the distribution of VE-cadherin, JAM-A and claudin-5 expression in hBMVEC monolayers (scale bar, 20 μm). (PC9-NC, PC9 cells transfected with negative control plasmid. PC9-OE, PC9 cells transfected with MSLN plasmid. PC9-BrM-NC, PC9-BrM cells transfected with negative control shRNA. PC9-BrM-SH1, PC9-BrM cells transfected with MSLN-targeted shRNA1. PC9-BrM-SH2, PC9-BrM cells transfected with MSLN-targeted shRNA2. PC9-BrM-SIR-1, PC9-BrM cells transfected with MET-targeted siRNA-1. PC9-BrM-SIR-2, PC9-BrM cells transfected with MET-targeted siRNA-2. PC9-BrM-SH2-NC, PC9-BrM-SH2 cells transfected with negative control plasmid. PC9-BrM-SH2-OE, PC9-BrM-SH2 cells transfected with MET plasmid. MET-SIR-1, PC9-BrM cells transfected with MET-targeted siRNA-1. shMSLN, PC9-BrM cells transfected with MSLN-targeted shRNA1. MET-OE, PC9-BrM cells transfected with MET plasmid.Data are presented as mean ± SD, ns, no significance)

Studies have shown that MET plays a biological role in the BM of many tumor cells [5, 32], including the ability of the cells to cross the BBB [33]. We assessed the co-expression of MSLN and MET in surgical specimens of primary lung tumors (LT, *n*=25) and brain metastases of lung cancer (BM, *n*=25), and found that both MSLN and MET were expressed at elevated levels in surgical specimens of brain metastases of lung cancer, and showed a close correlation (Fig. 4A-C). In NSCLC lines, western blot analysis further confirmed that the expression levels of MET and p-MET decreased with knockdown of MSLN, whereas overexpression of MSLN promoted the cellular expression levels of MET and p-MET (Fig. 4D-E, Fig. S5A). To determine whether MET is involved in MSLN-regulated tumor cell penetration of the BBB, we knocked down MET in PC9-BrM cells with siRNA (Fig. 4F) and then overexpressed MSLN in PC9-BrM cells in which we had previously knocked down MSLN (PC9-BrM-SH2) (Fig. 4G). *In vitro* and *ex vivo* trans-BBB assays showed that MET knockdown inhibited the ability of PC9-BrM cells to penetrate the BBB, whereas MET overexpression relieved the suppression of the trans-BBB ability of PC9-BrM-SH2 cells (Fig. 4H, Fig. S5B). We further evaluated expression of the junctional proteins of the BBB after treatment of hBMVECs with conditioned medium from the indicated metastatic cells. The results showed that MET knockdown inhibited the ability of brain metastatic cells to degrade TJ complexes, whereas overexpression of MET significantly enhanced the TJ complex degrading capacity of PC9-BrM-SH2 cells (Fig. 4I, J). Taken together, these data suggest that the effect of MSLN on tumor cell penetration of the BBB is dependent on MET.

### MSLN regulates the expression and phosphorylation of MET through the JNK signaling pathway in brain metastatic cells

**Fig. 5:**
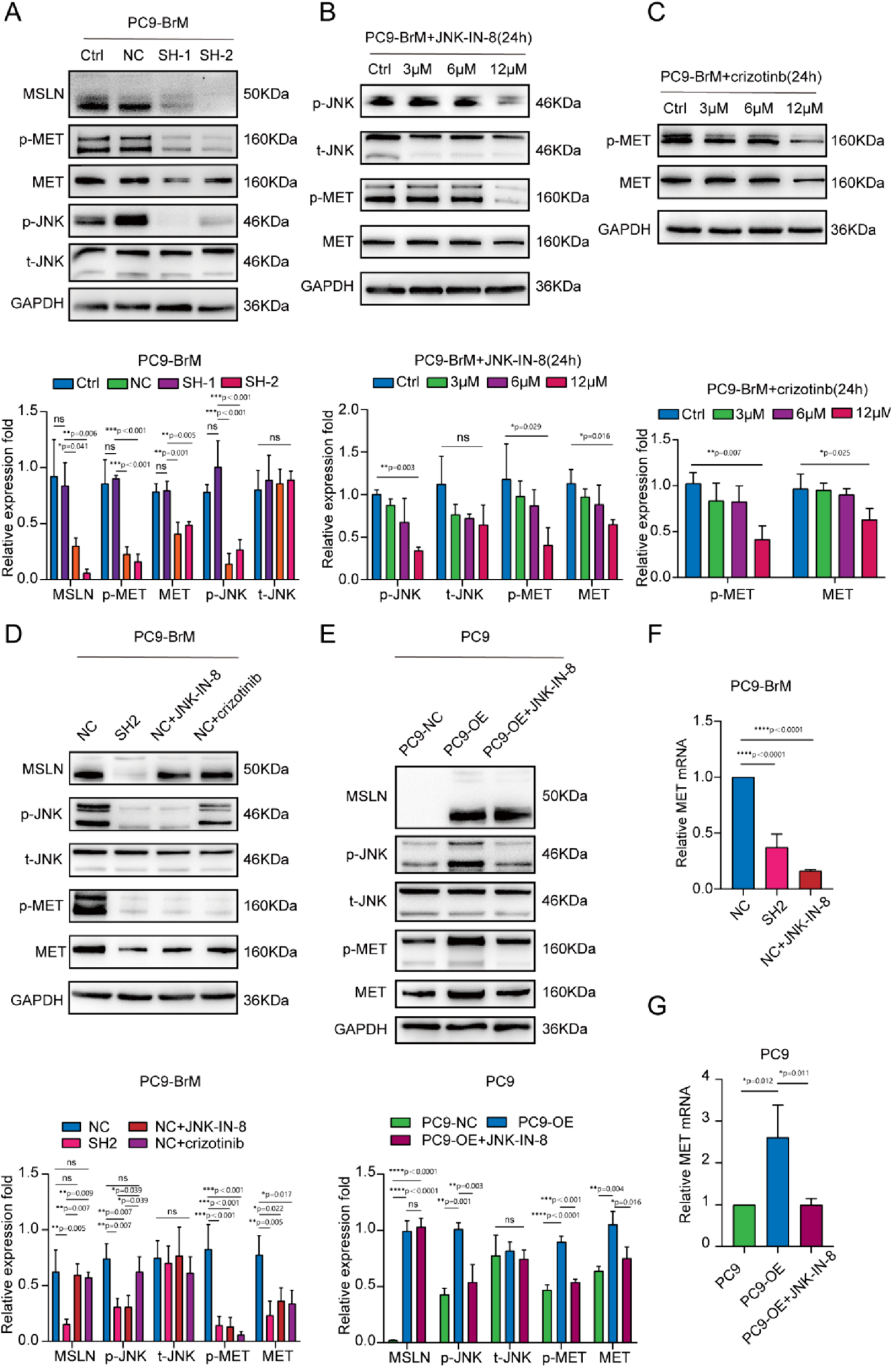
MSLN regulates MET phosphorylation as well as protein and mRNA expression in NSCLC cells through the JNK signaling pathway. **A** Representative western blot images showing MSLN, p-JNK, t-JNK, p-MET and MET expression levels after MSLN knockdown in PC9-BrM cells. **B, C** PC9-BrM cells were treated with JNK-IN-8 and crizotinib at different concentrations of 0, 3, 6, and 12 µM for 24 h, and the expression of the indicated molecules was detected by western blotting. **D-G** PC9-BrM-NC cells, PC9-BrM-SH2 cells, PC9-NC cells and PC9-OE cells were incubated in serum-free medium for 24 h, and then PC9-BrM-NC cells and PC9-OE cells were treated with the designated inhibitors at 12 µM for 24 h. The expression of the indicated molecules was then detected by western blotting and qRT-PCR. (PC9-NC, PC9 cells transfected with negative control plasmid. PC9-OE, PC9 cells transfected with MSLN plasmid. PC9-BrM-NC, PC9-BrM cells transfected with negative control shRNA. PC9-BrM-SH1, PC9-BrM cells transfected with MSLN-targeted shRNA1. PC9-BrM-SH2, PC9-BrM cells transfected with MSLN-targeted shRNA2. Data are presented as mean ± SD, ns, no significance)

Previous studies reported that activation of JNK promotes NSCLC metastasis by activating MMPs [34]. In addition, enhancement of JNK signaling promotes activation of MMP9, which further promotes the degradation of TJ proteins and leakage of the BBB [35]. To further determine the potential mechanisms underlying the effect of MSLN on MET expression, we detected JNK activity and MET expression in PC9-BrM cells transfected with MSLN-targeted shRNA or treated with a JNK inhibitor, JNK-IN-8, or a MET inhibitor, crizotinib. Western blot analysis demonstrated that both knockdown of MSLN and JNK-IN-8 treatment led to inhibited expression and phosphorylation of MET along with suppression of JNK activity in brain metastatic cells, whereas overexpression of MSLN significantly enhanced JNK activity in these cells (Fig. 5A-B, F, Fig. S5A, C). Notably, JNK-IN-8 treatment did not affect the MSLN expression but did efficiently attenuate MET activation in NSCLC cells overexpressing MSLN, whereas crizotinib had no effect on MSLN expression and JNK signaling (Fig. 5C-D, E-G). Taken together, these results suggest that MSLN regulates the expression and phosphorylation of MET through the JNK signaling pathway in brain metastatic cells.

### Targeting MSLN and MET therapeutically inhibits BM in vivo

**Fig 6:**
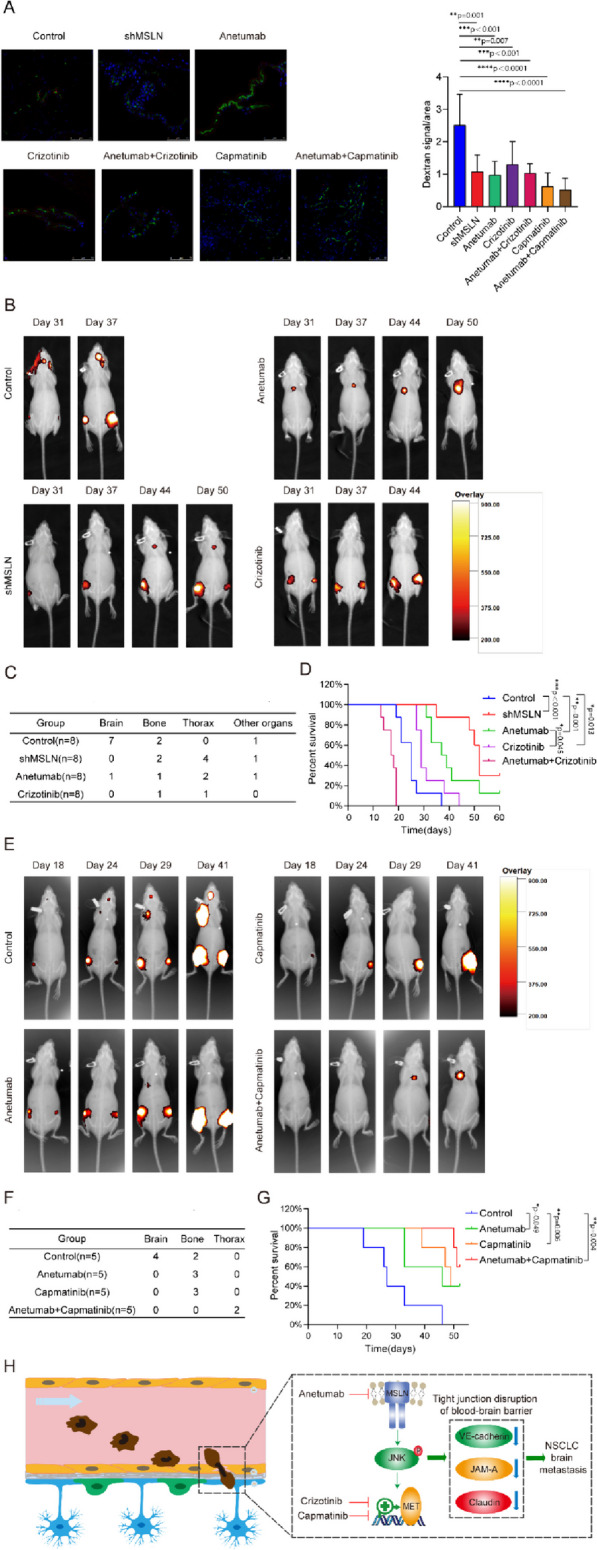
Targeting MSLN and MET therapeutically inhibits lung cancer BM *in vivo*. PC9-BrM cells and PC9-BrM cells with MSLN knockdown (shMSLN) were injected into nude mice via the left ventricle. Nude mice injected with PC9-BrM cells were randomly assigned to the following administration regimen groups and continued to be administered for 5 weeks from the day 3 post-injection: Control, placebo administration; anetumab, anetumab (0.2 mg/kg) intravenously weekly; crizotinib, crizotinib (5 mg/kg) intraperitoneally every 2 days; anetumab+crizotinib, anetumab (0.2 mg/kg) intravenously weekly and crizotinib (5 mg/kg) intraperitoneally every 2 days; capmatinib, oral capmatinib (10mg/kg) daily; anetumab+capmatinib, anetumab (0.2 mg/kg) intravenously weekly and oral capmatinib (10mg/kg) daily. **A** Fluorescent images showing BBB permeability of the mouse brains after intravenous injection of Texas Red-Dextran (70,000 MW), DyLight 488-Lycopersicon Esculentum Lectin (LEL) (*n*=3 in each group). Cell nuclei are stained with DAPI (blue). Scale bar, 75 μm. **B** Representative biofluorescence images of each group at the indicated time. **C** Results for distant metastasis in each group. **D** Survival curves for the different groups (*n*=8 in each group). **E** Representative biofluorescence images of each group at the indicated times. **F** Results for distant metastasis in each group.** G** Survival curves for the different groups (*n*=5 in each group). **H** Schematic description of the role of MSLN in promoting lung cancer BM by disrupting the BBB. (Data are presented as mean ± SD)

A previous study demonstrated that anetumab can specifically target MSLN-positive tumors and inhibit tumor growth in subcutaneous and orthotopic xenograft models [36], and another study reported that anetumab has preliminary anti-tumor activity in patients with MSLN-positive solid tumors in a phase I study [37]. The non-selective tyrosine kinase inhibitor (TKI) for MET crizotinib has been widely used in the clinical treatment of lung cancer patients including those with BM [38], and the selevtive MET-TKI capmatinib, which also inhibits the phosphorylation of MET in PC9-BrM cells (Fig. S6), has been recently approved and applied for NSCLC treatment [39]. We further evaluated the therapeutic efficacies of MSLN- or MET-targeting therapies in an *in vivo* preclinical BM model. For establishment of the mouse models, PC9-BrM cells (control) or PC9-BrM cells with MSLN knockdown (shMSLN) were introduced into nude mice by intracardiac injection. Anetumab, crizotinib and capmatinib were administered separately or in combination to mice inoculated with PC9-BrM cells. An *in vivo* BBB leakiness assay was performed by intravenous injection of Texas Red-Dextran (70,000 MW), DyLight 488-Lycopersicon Esculentum Lectin (LEL) on the 10th day. Dextran was used as an indicator of BBB leakiness while LEL was used to label the BBB. The diffused dextran indicated the impared BBB in mice injected by PC9-BrM cells (Control) while the dextran diffusion was significantly suppressed once MSLN and MET are targeted separately or jointly (Fig. 6A). As evidenced by the regular weekly bioluminescence images, it was found that anetumab, crizotinib and genetic silencing of MSLN all significantly inhibited the occurrence of BM *in vivo* and prolonged the survival of mice (Fig. 6B-D). Capmatinib also showed significantly inhibitory effect on the development of BM, and the improvement in the survival of mice was observed in the combined treatment group with capmatinib and anetumab (Fig. 6E-G). However, combined treatment with anetumab and crizotinib did not result in prolonged survival of the mice, with most mice dying within 20 days without any secondary metastases. Overall, our *in vivo* results suggest that therapies targeting MSLN and MET exhibited remarkable therapeutic efficacy for inhibiting the BM.

## Discussion

MSLN, a cell surface glycoprotein, is highly expressed in various tumor tissues [18, 28, 29, 40, 41], while it is found at very low levels in normal human tissues [10, 42]. A study showed that knockdown of MSLN significantly inhibits *in vitro* cell adhesion, migration, and invasion (critical steps necessary for metastasis), and also reverses EMT and attenuates stem cell properties in lung cancer cells [18]. In this study, we found that MSLN is not only a specific expression of the tumor antigen in situ in lung cancer compared to paracancer, but its expression is further elevated in brain metastases. This means that MSLN may play an important role in the pathological process of BM. Since soluble MSLN in serum samples may also be a potential serum biomarker for malignancies [26], we also found that the MSLN level of the ELC group was higher than that of the HC group, and the MSLN level in the LCBM group was even higher than that of the ELC group, indicating the important significance of MSLN in NSCLC that different threshold expression levels can help to diagnose primary lung cancer and secondary BM and predict the risk of lung cancer BM. In addition, serum levels of MSLN were found to be significantly higher in the LCBM group than in the PBT group. Since there is no evidence that MSLN is a tumor antigen in PBT, this finding suggests that serum MSLN may be useful in identifying primary and secondary intracranial tumors. Therefore, MSLN is a good indicator of NSCLC progression, especially for BM.

Tumor cell penetration of the BBB is the rate-limiting step in BM [43, 44]. To study the pathology of BM, we used both a conventional Transwell model and our constructed multi-organ microfluidic chip to study tumor cell extravasation across the BBB. The 'BBB' constructed on the chip mimics the physiological microenvironment in terms of structural integrity and barrier function, and allows real-time visualisation of the entire tumor BM process, which is not possible with the Transwell system or animal models [22]. Our experiments with both models have shown that MSLN promotes the crossing of the BBB by NSCLC cells. BBB disruption is necessary for tumor cell migration across the endothelium and is achieved by degradation of brain endothelial cell junction proteins. We treated brain endothelial cells with conditioned medium from brain metastatic cells and found that knockdown of MSLN inhibited the degradation of VE-cadherin, JAM-A and claudin-5 on brain endothelial cells, significantly reducing the number of tumor cells migrating across the endothelial layer.

With the development of prevention and evidence-based medicine, it has been considered more important to prevent metastasis than to treat it. Based on our findings that MSLN promotes BM by encouraging NSCLC cells to cross the BBB, a key rate-limiting link, targeting MSLN is expected to be a therapeutic strategy for preventing BM in NSCLC. As MSLN expression is rather low in most normal tissues, but highly elevated in tumors, the current main strategies for targeting MSLN include tumor vaccines, antibody-based therapies and chimeric antigen receptor T-cell (CAR-T) therapies. The combination of the bacterial vaccine CRS-207, an attenuated form of a *Listeria* monocytogenes vector overexpressing human MSLN, with pemetrexed/cisplatin chemotherapy provided objective disease control in unresectable malignant pleural mesothelioma and induced significant clinical responses, suggesting that tumor vaccines may be potential candidates for cancer therapy [45]. Anetumab ravtansine (ARav) is a novel antibody-drug conjugate currently in clinical trials for several malignancies that express MSLN. The antibody binds MSLN with high affinity and induces internalisation of DM4 (the conjugate combines with ravtansine). Once inside the cell, the SPDB (N-succinimidyl 4-(2-pyridyldithio)butnoate) junction is cleaved [46] and DM4 binds to microtubule proteins, disrupting microtubule dynamics and thereby inhibiting cell division and proliferation. *In vivo*, ARav showed potent anti-tumor activity against MSLN-expressing mesothelioma, pancreatic and ovarian xenografts from cancer patients [36]. Among immunotherapies, CAR-T therapy is considered one of the most promising new approaches for cancer treatment. CAR-T cells are engineered T cells that produce an artificial T receptor targeting a specific protein. To date, fourth-generation CARs favor the secretion of cytokines (including IL-12 and IL-15) and thus strongly influence the immune components of the tumor microenvironment [47]. Preclinical studies in a mouse model of metastatic pancreatic adenocarcinoma demonstrated that CAR-T cells targeting MSLN can induce tumor cytotoxicity and eradicate lung metastases [48, 49]. In an *in situ* mouse model of mesothelioma, local intrapleural injection of CAR-T cells targeting MSLN produced potent anti-tumor activity that correlated with their proliferation and persistence after 200 days [50]. Similar results were recently reported in a preclinical model of gastric cancer following peritumor injection of CAR-T cells targeting MSLN [51]. In the present study, we found that anetumab reduced the incidence of lung cancer BM and effectively prolonged the survival of mice. These results provide support for the further investigation of MSLN-targeted therapy in patients with NSCLC BM.

In this study, we further elucidated the mechanism by which MSLN promotes NSCLC cell trans-BBB and found that MSLN-mediated BBB disruption by NSCLC cells is dependent on MET expression and activation. In NSCLC, the three main mechanisms of MET dysregulation include protein overexpression, MET exon 14 jump mutation (METex14) or gene amplification. The MET protein encoded by the MET gene is a tyrosine kinase receptor. Upon activation, MET dimerisation and tyrosine phosphorylation occur, which activates downstream signalling pathways such as PI3K/AKT, RAS/MAPK, STAT and Wnt/β-catenin, etc., which promote the survival, proliferation, invasion and drug resistance in lung cancer [52]. MET knockdown was found to significantly reduce the incidence of BM from NSCLC cells *in vitro* [5]. Increased plasma soluble Met (sMet) levels are associated with lower overall survival in NSCLC patients [53], supporting the results of other studies which showed that MET overexpression and amplification are associated with poor prognosis in NSCLC patients [54–57]. As a result, capmatinib, a selective MET inhibitor, was approved by the FDA recently. In a clinical trial, the combination of capmatinib with EGFR-TKIs is determined as a promising treatment option for patients with EGFR-mutated, MET-dysregulated NSCLC and particularly for patients with MET-amplified tumors [58]. Capmatinib showed a clinically meaningful rate of anti-tumor activity and an acceptable safety profile in pretreated advanced NSCLC patients with either MET gene copy number (GCN) ≥6 and/or METex14 mutation [59]. Crizotinib, an FDA-approved small molecule inhibitor of the ALK, MET and ROS1 tyrosine kinases for advanced NSCLC [60–63], has shown satisfactory antitumor activity [64]. A recent study has reported the sensitivity to crizotinib-targeted therapy in patients with BM from NSCLC with concomitant activation of MET receptors and ALK fusion genes [65]. In the present study, we found that crizotinib and capmatinib significantly inhibited the occurrence of BM *in vivo* and prolonged the survival of the mice. Noteworthy, animal studies indicated that combination of crizotinib and anetumab lead to shorter survival while combination of capmatinib and anetumab showed a better efficiency. We hypothesized that crizotinib may cause more toxic side effects since it does not only target the MET. Moreover, *in vivo* tolerance to the combination of crizotinib and anetumab needs to be further explored. Taken together, these data support the potential targeted use of the MET selective TKI capmatinib or the MET non-selective TKI crizotinib according to the driver gene characteristics of patients with advanced NSCLC to provide preventive strategies for BM.

## Conclusions

Our study provides evidence that MSLN promotes MET expression and activation via the JNK signalling pathway, which helps tumor cells degrade TJs of the BBB, thereby promoting the development of BM. MSLN can be used not as a biomarker for the diagnosis and prognosis of NSCLC, but also as an effective target for the therapies for patients with BM. Application of MSLN-targeted inhibitor (anetumab) or MET-targeted inhibitors (crizotinib/capmatinib) provides new preventive strategies for NSCLC BM (Fig. 6H).

## Limitation

There remain some possible limitations in this study. First, as our clinical samples are from a single center, the sample size is limited. Future multicenter and large-scale studies are warranted to further verify the conclusions. Secondly, our *in vivo* and *in vitro* studies were mainly conducted by using lung adenocarcinoma cell lines. Squamous and large cell lung cancer cell lines with brain metastasis characteristics need to be established in the future. Finally, our *in vivo* experiments were conducted in nude mice which excluded the regulation of MSLN on immunity and its effect on BM outcomes. It is also unknown whether the drugs will induce immune-related responses *in vivo* which may thus affect the therapeutic efficiency.

### Supplementary Information

**Supplementary Material 1.** 

**Supplementary Material 2.** 

**Supplementary Material 3.**

**Supplementary Material 4.** 

**Supplementary Material 5.** 

## Data Availability

All data are available from the Prof. Qi Wang upon reasonable request.

## Abbreviations

BM: Brain metastasis
NSCLC: Non-small cell lung cancer
MSLN: Mesothelin
BBB: Blood-brain barrier
JNK: c-Jun N-terminal kinase
HGFR: Hepatocyte growth factor receptor
RTK: Transmembrane receptor tyrosine kinase
p-MET: Phosphorylated MET
FDA: Food and Drug Administration
ALK: Anaplastic lymphoma kinase
TJs: Tight junctions
EMT: Epithelial-mesenchymal transition
16HBE cells: Human bronchial epithelial cells
hPMECs: Human lung microvascular endothelial cells
HFL1 cells: Human lung fibroblasts
THP-1 cells: Human monocyte cells
BEAS-2B cells: Normal bronchial epithelial cells
hBMVECs: Human brain microvascular endothelial cells
HA-1800: Human astrocytes
RIPA: Radioimmunoprecipitation assay
SDS: Sodium dodecyl sulfate
PAGE: Polyacrylamide gel electrophoresis
MMP7: Matrix metalloproteinase 7
GAPDH: Glyceraldehyde phosphate dehydrogenase
VE: Vascular endothelial
TBST: Tris-buffered saline with Tween
ECL: Enhanced chemiluminescence
ELISA: Enzyme-linked immunosorbent assay
OD: Optical density
HRP: Horse radish peroxidase
DAB: Diaminobenzidine
PBS: Phosphate-buffered saline
GFP: Green fluorescent protein
IF: Immunofluorescence
BSA: Bovine serum albumin
FITC: Fluorescein isothiocyanate
DAPI: 4',6-diamidino-2-phenylindole
qRT-PCR: Quantitative reverse transcription-polymerase chain reaction
SD: Standard deviation
SPSS: Statistical Package for the Social Sciences
ANOVA: Analysis of variance
PBT: Primary brain tumors
HC: Healthy controls
ELC: Early lung cancer
BoM: Bone metastasis
LM: Liver metastasis
LCBM: Lung cancer brain metastasis
EGFR: Epidermal growth factor receptor
MMPs: Matrix metalloproteinase family members
JAMs: Junctional adhesion molecules
LT: Lung tumor
TKI: Tyrosine kinase inhibitor
LEL: Lycopersicon Esculentum Lectin
CAR-T: Chimeric antigen receptor T-cell
Arav: Anetumab ravtansine
DM4: The conjugate combines with ravtansine
SPDB: N-succinimidyl 4-(2-pyridyldithio)butnoate
sMet: Soluble Met
GCN: Gene copy number
ROS1: ROS proto-oncogene 1, receptor tyrosine kinase
PI3K: Phosphoinositide 3-kinase
MAPK: Mitogen-activated protein kinase
STAT: Signal transducer and activator of transcription
METex14: MET exon 14 jump mutation
