# Supplementary material for: Mesothelin promotes brain metastasis of non-small cell lung cancer by activating MET
Source: J Exp Clin Cancer Res. 2024 Apr 3;43:103. doi: 10.1186/s13046-024-03015-w (PMC10988939; doi:10.1186/s13046-024-03015-w)
Supplement: Supplementary file 2 — Supplementary Material 2. [file 13046_2024_3015_MOESM2_ESM.docx]

**Table S2.** Correlation between MSLN expression and clinicopathological parameters in patients with lung cancer.

| Clinicopathological parameter | Variables | MSLN expression | | Total | χ2 | *p* value |
| --- | --- | --- | --- | --- | --- | --- |
|  |  | Low | High |  |  |  |
| Age |  |  |  |  | 0.109 | 0.742 |
|  | ＜60 | 16 | 9 | 25 |  |  |
|  | ≥60 | 27 | 18 | 45 |  |  |
| Gender |  |  |  |  | 0.273 | 0.601 |
|  | Female | 17 | 9 | 26 |  |  |
|  | Male | 26 | 18 | 44 |  |  |
| Smoke |  |  |  |  | 2.439 | 0.295 |
|  | Without | 31 | 16 | 47 |  |  |
|  | With | 12 | 10 | 22 |  |  |
|  | Null | 0 | 1 | 1 |  |  |
| Histology |  |  |  |  | 0.120 | 0.729 |
|  | Adenocarcinoma | 37 | 24 | 61 |  |  |
|  | Squamous | 6 | 3 | 9 |  |  |
| T stage |  |  |  |  | 0.498 | 0.480 |
|  | T1/T2 | 39 | 23 | 62 |  |  |
|  | T3/T4 | 4 | 4 | 8 |  |  |
| N stage |  |  |  |  | 4.384 | 0.112 |
|  | N0 | 11 | 4 | 15 |  |  |
|  | N1/N2/N3 | 23 | 21 | 44 |  |  |
|  | Null | 9 | 2 | 11 |  |  |
| M stage |  |  |  |  | 46.539 | <0.001^***^ |
|  | M0 | 36 |  | 36 |  |  |
|  | M1 | 7 | 27 | 34 |  |  |
|  | Null |  |  |  |  |  |
| TNM stage |  |  |  |  | 32.965 | <0.001^***^ |
|  | I/II | 30 |  | 30 |  |  |
|  | III/IV | 13 | 27 | 40 |  |  |

^*^*P*<0.05, ^**^*P*<0.01, ^***^ *P*<0.001
