# Supplementary material for: Mesothelin promotes brain metastasis of non-small cell lung cancer by activating MET
Source: J Exp Clin Cancer Res. 2024 Apr 3;43:103. doi: 10.1186/s13046-024-03015-w (PMC10988939; doi:10.1186/s13046-024-03015-w)
Supplement: Supplementary file 3 — Supplementary Material 3. [file 13046_2024_3015_MOESM3_ESM.docx]

**Table S3.** Clinicopathologic imaging features of the serum cohort.

| Characteristics  stics | Case | Characteristics | Case |
| --- | --- | --- | --- |
| Lung cancer brain metastasis | 42 | Controls | 112 |
| Gender |  | Gender |  |
| Male | 20 | Male | 54 |
| Female | 22 | Female | 58 |
| Age (Year) |  | Age (Year) |  |
| ≤60 | 20 | ≤60 | 67 |
| >60 | 22 | >60 | 45 |
| Other organ metastasis |  | Type |  |
| Without | 22 | NSCLC | 65 |
| With | 20 | Glioma | 23 |
|  |  | Healthy group | 24 |
| Pathological type |  | Pathological type |  |
| Adenocarcinoma | 35 | Adenocarcinoma | 58 |
| Squamous cell carcinoma | 6 | Squamous cell carcinoma | 6 |
| Other types | 1 | Other types | 1 |
| Number of brain metastatic lesions |  | TNM stage |  |
| 1 | 21 | I / II | 22 |
| ≥2 | 21 | III / IV | 43 |
| Maximum diameter of BM (cm) |  | Organ metastasis |  |
| ≤2 | 29 | Liver | 20 |
| >2 | 13 | Bone | 23 |
| Meningeal metastasis |  | M stage |  |
| Without | 33 | M0 | 22 |
| With | 9 | M1 | 43 |
| T stage |  | T stage |  |
| T1 | 5 | T1 | 30 |
| T2 | 14 | T2 | 13 |
| T3 | 7 | T3 | 7 |
| T4 | 16 | T4 | 15 |
| N stage |  | N stage |  |
| N0 | 6 | N0 | 29 |
| N1 | 1 | N1 | 5 |
| N2 | 18 | N2 | 19 |
| N3 | 17 | N3 | 12 |
