# Supplementary material for: Mesothelin promotes brain metastasis of non-small cell lung cancer by activating MET
Source: J Exp Clin Cancer Res. 2024 Apr 3;43:103. doi: 10.1186/s13046-024-03015-w (PMC10988939; doi:10.1186/s13046-024-03015-w)
Supplement: Supplementary file 4 — Supplementary Material 4. [file 13046_2024_3015_MOESM4_ESM.docx]

**Table S4.** Correlation analysis of MSLN expression and clinicopathological imaging features in serum validated cohort.

| Characteristics | Case | MSLN_*P* | Characteristics | Case | MSLN_*P* |
| --- | --- | --- | --- | --- | --- |
| Gender |  |  | Number of lung primary lesions | | |
| Male | 20 (47.62%) | 0.455 | 1 | 18 (42.86%) | 0.020^*^ |
| Female | 22(52.38%) |  | ≥2 | 24 (57.14%) |  |
| Age (Year) |  |  | Maximum diameter of LP (cm) | |  |
| ≤60 | 20 (47.62%) | 0.347 | ≤2 | 17 (40.48%) | 0.735 |
| >60 | 22 (52.38%) |  | >2 | 25 (59.52%) |  |
| Pathological type | | 0.875 | Pleural effusion |  |  |
| Adenocarcinoma | 35 (83.33%) |  | Without | 26 (61.90%) | ＜0.001^***^ |
| Squamous cell carcinoma | 6(14.29%) |  | With | 16 (38.10%) |  |
| Other types | 1 (2.38%) |  | Lung surgery |  |  |
| Smoke |  |  | Without | 32 (76.19%) | 0.243 |
| Without | 30 (71.43%) | 0.023^*^ | With | 10 (23.81%) |  |
| With | 12 (28.57%) |  | T stage |  |  |
| Other organ metastasis | | | T1 | 5 (11.90%) | 0.758 |
| Without | 22 (52.38%) | 0.379 | T2 | 14 (33.33%) |  |
| With | 20 (47.62%) |  | T3 | 7 (16.67%) |  |
| Number of brain metastatic lesions | | | T4 | 16 (38.10%) |  |
| 1 | 21 (50.00%) | 0.540 | N stage |  |  |
| ≥2 | 21 (50.00%) |  | N0 | 6 (14.29%) | 0.645 |
| Maximum diameter of BM (cm) | | | N1 | 1 (2.38%) |  |
| ≤2 | 29 (69.05%) | 0.003^**^ | N2 | 18 (42.86%) |  |
| >2 | 13 (30.95%) |  | N3 | 7 (16.67%) |  |
| Meningeal metastasis | | | EGFR |  |  |
| Without | 33 (78.57%) | 0.020^*^ | Wild type | 8 (27.59%) | ＜0.001^***^ |
| With | 9 (21.43%) |  | Mutant | 21 (72.41%) |  |

^*^*P*<0.05, ^**^*P*<0.01, ^***^ *P*<0.001
