# Supplementary material for: Mesothelin promotes brain metastasis of non-small cell lung cancer by activating MET
Source: J Exp Clin Cancer Res. 2024 Apr 3;43:103. doi: 10.1186/s13046-024-03015-w (PMC10988939; doi:10.1186/s13046-024-03015-w)
Supplement: Supplementary file 5 — Supplementary Material 5. [file 13046_2024_3015_MOESM5_ESM.docx]

**Supplementary Figures
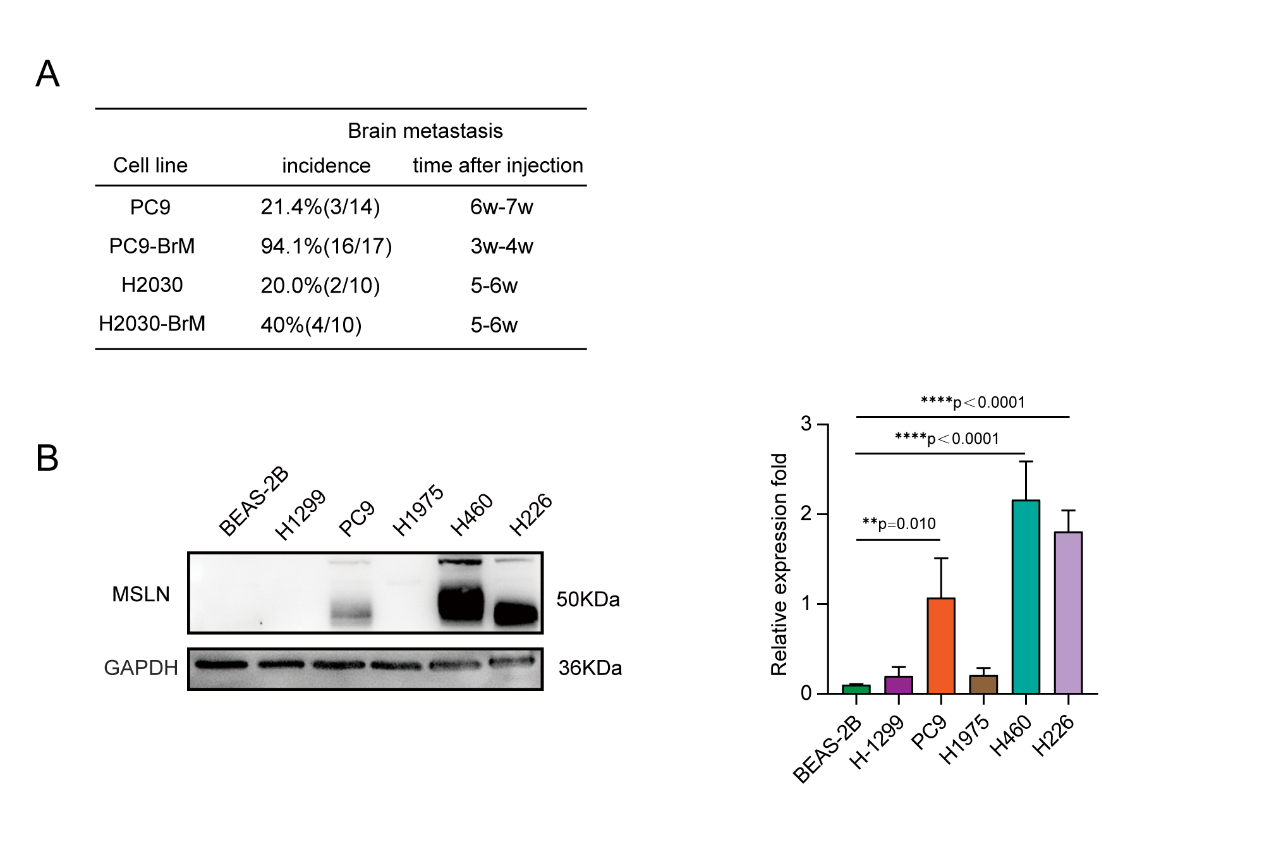
**

**Supplementary Figure1.** **MSLN is highly expressed in NSCLC cell lines with high brain metastatic potential. A** Frequency and time circle of BM after inoculation of indicated cells in nude mice. **B** the expression of MSLN in normal bronchial epithelial cells (BEAS-2B) and lung cancer cell lines (H1299, PC9, H1975, H460, H226). (Data are presented as mean ± SD)


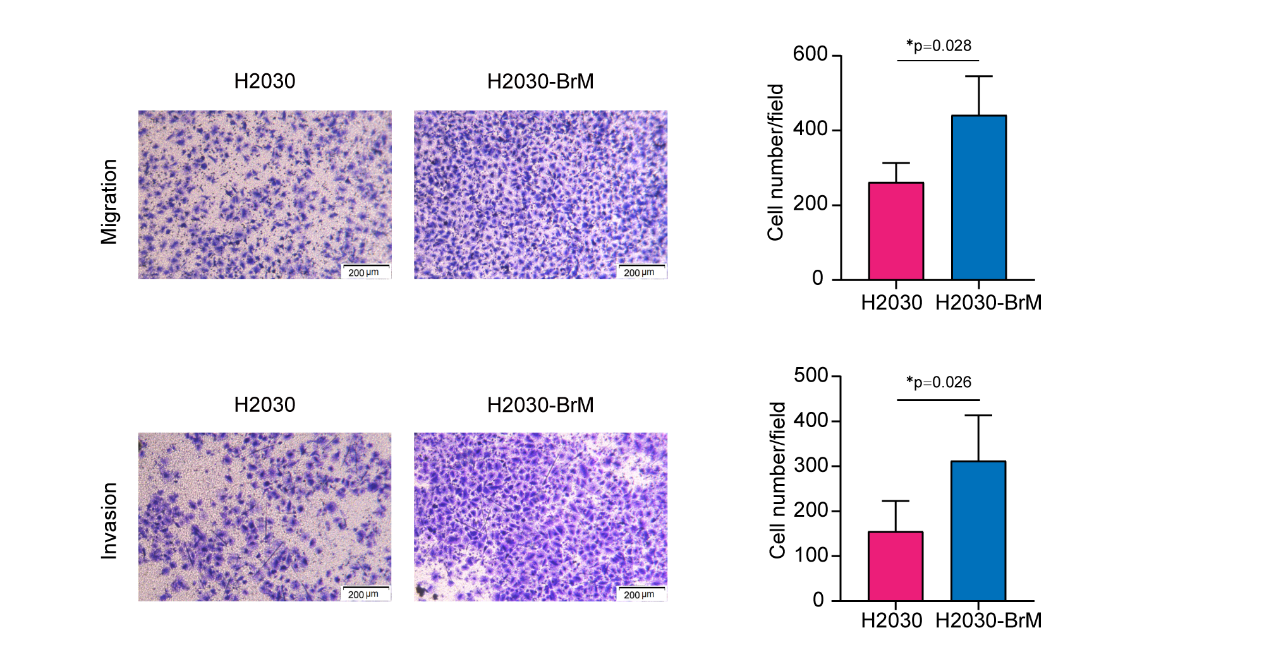


**Supplementary Figure2. Highly-brain metastatic cells have stronger migration and invasion ability compared to parental cells.** Transwell migration and invasion assays to determine the effect of altered MSLN expression on the migration and invasion of lung adenocarcinoma cells(scale bar, 200μm). (Data are presented as mean ± SD)


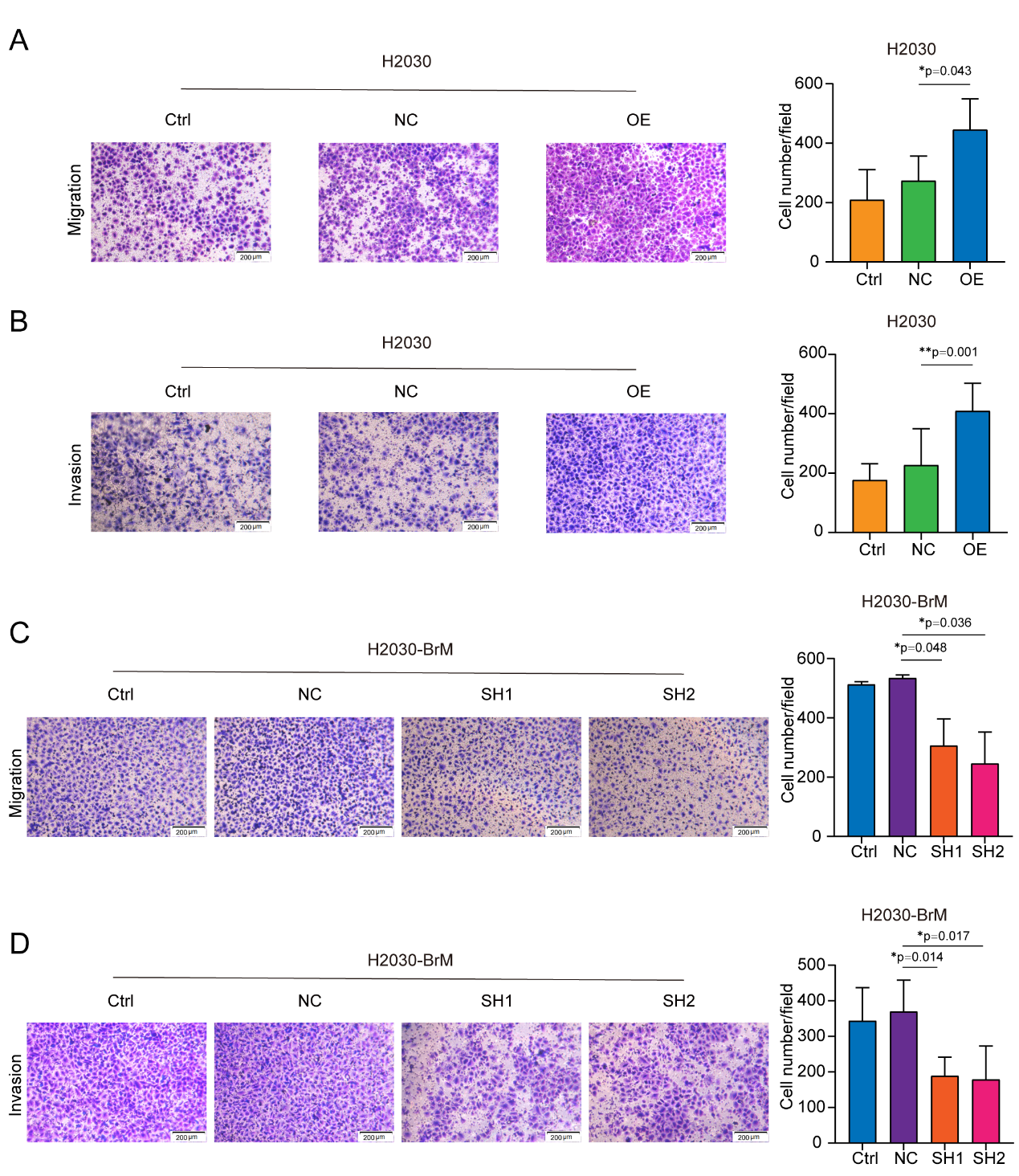


**Supplementary Figure3. MSLN promotes the migration and invasion of H2030 and H2030-BrM cells. A,B** Transwell migration and invasion assays to determine the effect of overexpressing MSLN expression on the migration and invasion of H2030 cells(scale bar, 200μm). **C,D** Transwell migration and invasion assays to determine the effect of MSLN knockdown on the migration and invasion of H2030-BrM cells(scale bar, 200μm). (H2030-NC, H2030 cells transfected with negative control plasmid. H2030-OE, H2030 cells transfected with MSLN plasmid. H2030-BrM-NC, H2030-BrM cells transfected with negative control shRNA. H2030-BrM-SH1, H2030-BrM cells transfected with MSLN-targeted shRNA1. H2030-BrM-SH2, H2030-BrM cells transfected with MSLN-targeted shRNA2.Data are presented as mean ± SD)


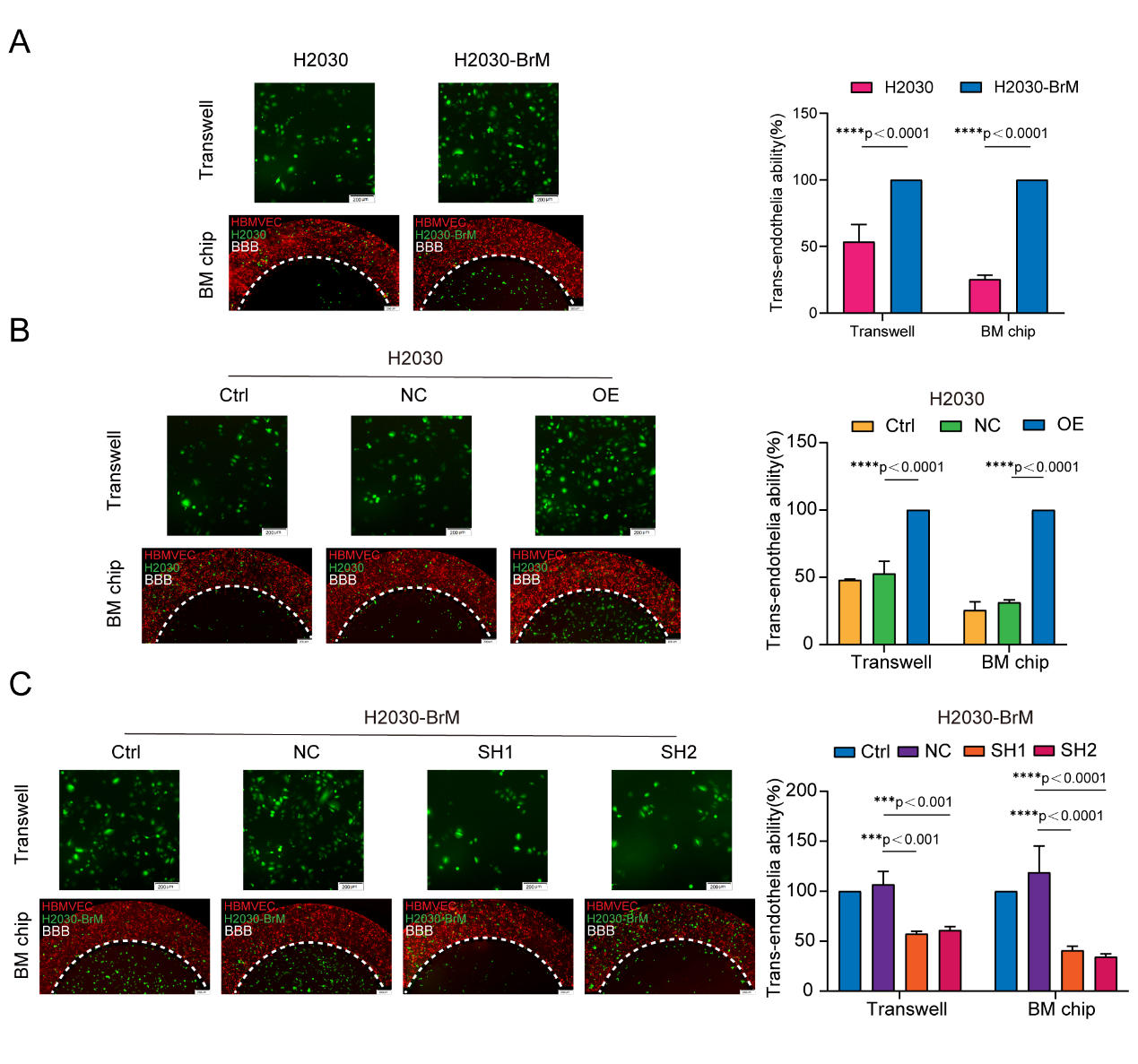


**Supplementary Figure4. MSLN helps H2030 and H2030-BrM cells penetrate the endothelium. A** Representative images show the ability of H2030 and H2030-BrM cells to penetrate the endothelium in a classic in vitro blood-brain barrier model(scale bar, 200μm). **B** In vitro assay of the effect of MSLN overexpression on the ability of H2030 cells to penetrate the endothelium(scale bar, 200μm). **C** The effect of MSLN knockdown on the ability of H2030-BrM cells to penetrate the endothelium was determined in vitro(scale bar, 200μm). (H2030-NC, H2030 cells transfected with negative control plasmid. H2030-OE, H2030 cells transfected with MSLN plasmid. H2030-BrM-NC, H2030-BrM cells transfected with negative control shRNA. H2030-BrM-SH1, H2030-BrM cells transfected with MSLN-targeted shRNA1. H2030-BrM-SH2, H2030-BrM cells transfected with MSLN-targeted shRNA2.Data are presented as mean ± SD)


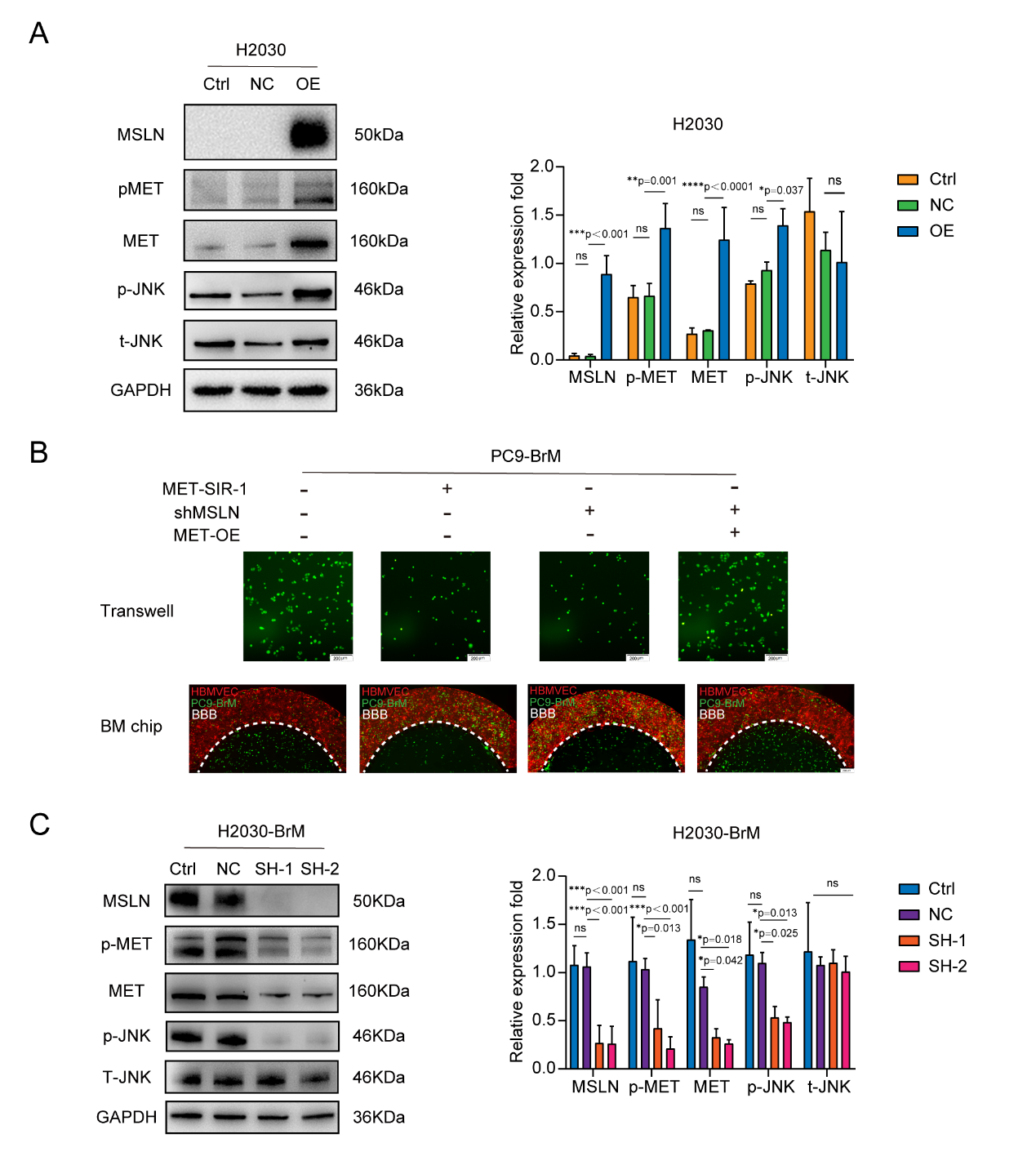


**Supplementary Figure5. MSLN promotes Highly-brain metastatic cells crossing of the BBB dependent on MET. A** Representative western blot images and quantitative results showed the expression levels of MSLN, p-JNK, t-JNK, p-MET and MET in H2030 cells after MSLN overexpression. **B** Representative images showing the effect of MET on the ability of PC9-BrM cells to penetrate the endothelium(scale bar, 200μm). **C** Representative western blot images and quantitative results showing MSLN, p-JNK, t-JNK, p-MET and MET expression levels after MSLN knockdown in H2030-BrM cells. (H2030-NC, H2030 cells transfected with negative control plasmid. H2030-OE, H2030 cells transfected with MSLN plasmid. H2030-BrM-NC, H2030-BrM cells transfected with negative control shRNA. H2030-BrM-SH1, H2030-BrM cells transfected with MSLN-targeted shRNA1. H2030-BrM-SH2, H2030-BrM cells transfected with MSLN-targeted shRNA2. MET-SIR-1, PC9-BrM cells transfected with MET-targeted siRNA-1. shMSLN, PC9-BrM cells transfected with MSLN-targeted shRNA1. MET-OE, PC9-BrM cells transfected with MET plasmid. Data are presented as mean ± SD)


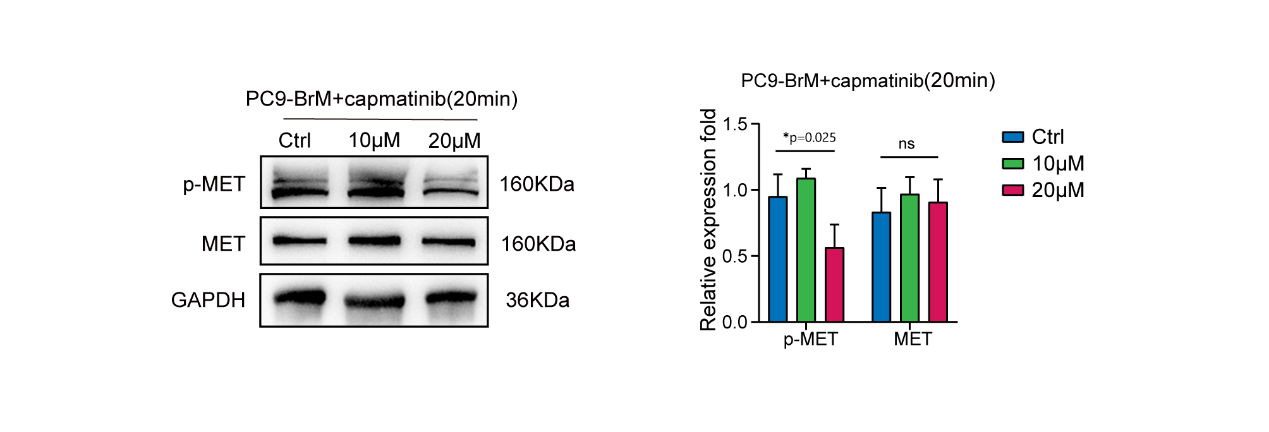


**Supplementary Figure6.** **Capmatinib inhibits the phosphorylation of MET in PC9-BrM cells.** PC9-BrM cells were treated with capmatinib at different concentrations of 0, 10, and 20 µM for 20 min, and the expression of the indicated molecules was detected by western blotting. (Data are presented as mean ± SD, ns: no significance)
